# Supplementary material for: The Influence of Short-Term Weather Parameters and Air Pollution on Adolescent Airway Inflammation
Source: Int J Environ Res Public Health. 2023 Sep 25;20(19):6827. doi: 10.3390/ijerph20196827 (PMC10572171; doi:10.3390/ijerph20196827)
Supplement: Supplementary file 1 [file ijerph-20-06827-s001.zip › ijerph-2527150-supplementary.docx]

**Supplementary material**


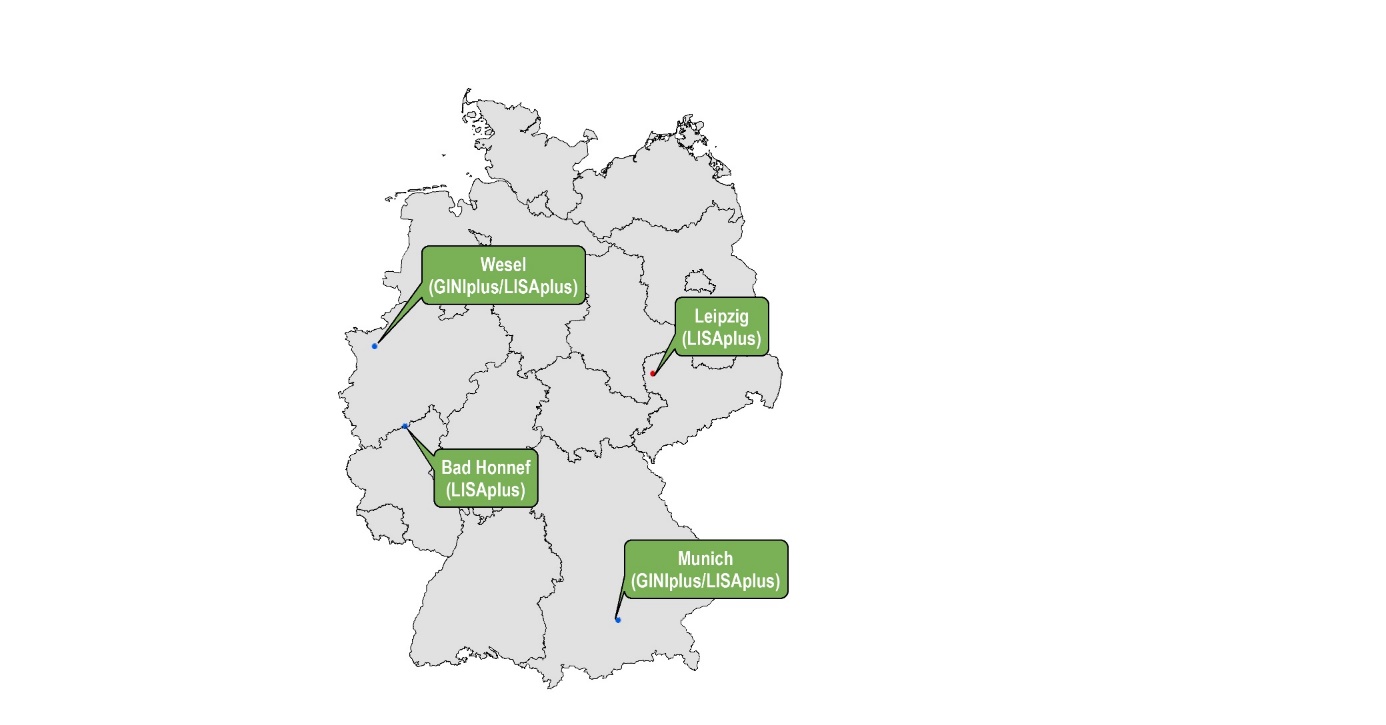


**Figure S1.** A map showing the study areas of the GINIplus and LISA birth cohorts in Germany.

**Table S1.** A table showing the main effects of relative humidity (RH), air pollution, and temperature on FeNO in German adolescents when stratified by sex.

|  | Female | | Male | |
| --- | --- | --- | --- | --- |
|  | Percentage change (95% CI)^2*^ | p-value^1*^ | Percentage change (95% CI)^2*^ | p-value^1*^ |
| RH | -0.01 (-0.03, 0.01) | 0.232 | -0.01 (-0.03, 0.02) | 0.586 |
| PM_2.5_ | 0.07 (-0.50, 0.64) | 0.814 | 0.20 (-0.43, 0.84) | 0.526 |
| Tmax | 0.18 (-0.55, 0.91) | 0.632 | 0.57 (-0.28, 1.42) | 0.192 |
| RH | -0.02 (-0.03, 0.00) | 0.085 | -0.01 (-0.03, 0.01) | 0.263 |
| PM_2.5_ | 0.07 (-0.50, 0.64) | 0.814 | 0.23 (-0.40, 0.87) | 0.477 |
| Tmin | 0.01 (-0.86, 0.89) | 0.982 | 0.74 (-0.27, 1.75) | 0.151 |
| RH | -0.01 (-0.03, 0.01) | 0.166 | -0.01 (-0.03, 0.01) | 0.459 |
| PM_2.5_ | 0.07 (-0.50, 0.64) | 0.805 | 0.22 (-0.41, 0.85) | 0.503 |
| Tmean | 0.17 (-0.66, 0.99) | 0.694 | 0.66 (-0.29, 1.62) | 0.172 |
| RH | -0.01 (-0.04, 0.01) | 0.235 | 0.00 (-0.03, 0.02) | 0.740 |
| O_3_ | -0.04 (-0.29, 0.22) | 0.782 | 0.06 (-0.21, 0.33) | 0.671 |
| Tmax | 0.22 (-0.54, 0.99) | 0.567 | 0.55 (-0.35, 1.45) | 0.232 |
| RH | -0.02 (-0.04, 0.00) | 0.129 | -0.01 (-0.03, 0.01) | 0.457 |
| O_3_ | -0.01 (-0.26, 0.24) | 0.907 | 0.06 (-0.20, 0.33) | 0.651 |
| Tmin | 0.02 (-0.88, 0.94) | 0.959 | 0.69 (-0.35, 1.74) | 0.194 |
| RH | -0.01 (-0.04, 0.01) | 0.181 | -0.01 (-0.03, 0.02) | 0.634 |
| O_3_ | -0.03 (-0.28, 0.22) | 0.811 | 0.06 (-0.21, 0.33) | 0.668 |
| Tmean | 0.20 (-0.66, 1.07) | 0.643 | 0.64 (-0.35, 1.64) | 0.209 |
| RH | -0.01 (-0.03, 0.01) | 0.218 | -0.01 (-0.03, 0.02) | 0.495 |
| NO_2_ | 0.20 (-0.32, 0.72) | 0.459 | 0.31 (-0.25, 0.86) | 0.279 |
| Tmax | 0.21 (-0.52, 0.94) | 0.577 | 0.65 (-0.20, 1.51) | 0.136 |
| RH | -0.02 (-0.03, 0.00) | 0.073 | -0.01 (-0.03, 0.01) | 0.172 |
| NO_2_ | 0.19 (-0.33, 0.72) | 0.474 | 0.35 (-0.21, 0.91) | 0.227 |
| Tmin | 0.05 (-0.83, 0.94) | 0.909 | 0.86 (-0.15, 1.89) | 0.097 |
| RH | -0.01 (-0.03, 0.01) | 0.151 | -0.01 (-0.03, 0.01) | 0.363 |
| NO_2_ | 0.20 (-0.32, 0.72) | 0.455 | 0.32 (-0.23, 0.88) | 0.256 |
| Tmean | 0.20 (-0.62, 1.03) | 0.633 | 0.77 (-0.19, 1.74) | 0.151 |
| ^1^ p-value <0.050 in bold  ^2^ per 5% increase in RH at Lag01  ^*^Adjusted for study location, season, chronic respiratory disease | | | | |

**Table S2.** A table showing the main effects of relative humidity (RH), air pollution, and temperature on FeNO in German adolescents when stratified by BMI.

|  | Underweight | | Normal Weight | | Overweight | |
| --- | --- | --- | --- | --- | --- | --- |
|  | Percentage change (95% CI)^2*^ | p-value^1*^ | Percentage change (95% CI)^2*^ | p-value^1*^ | Percentage change (95% CI)^2*^ | p-value^1*^ |
| RH | -0.02 (-0.05, 0.02) | 0.404 | -0.01 (-0.03, 0.01) | 0.511 | -0.02 (-0.07, 0.03) | 0.485 |
| PM_2.5_ | -0.11 (-1.11, 0.91) | 0.837 | 0.34 (-0.17, 0.85) | 0.194 | -0.18 (-1.52, 1.19) | 0.801 |
| Tmax | 0.15 (-1.20, 1.52) | 0.830 | 0.29 (-0.37, 0.95) | 0.392 | 0.64 (-1.21, 2.51) | 0.502 |
| RH | -0.02 (-0.05, 0.01) | 0.313 | -0.01 (-0.03, 0.01) | 0.233 | -0.03 (-0.07, 0.01) | 0.203 |
| PM_2.5_ | -0.09 (-1.10, 0.93) | 0.857 | 0.35 (-0.16, 0.86) | 0.182 | -0.18 (-1.53, 1.20) | 0.802 |
| Tmin | 0.37 (-1.22, 1.99) | 0.652 | 0.22 (-0.57, 1.01) | 0.586 | 0.26 (-1.86, 2.43) | 0.811 |
| RH | -0.02 (-0.05, 0.02) | 0.381 | -0.01 (-0.02, 0.01) | 0.405 | -0.02 (-0.07, 0.02) | 0.347 |
| PM_2.5_ | -0.10 (-1.11, 0.91) | 0.842 | 0.34 (-0.17, 0.86) | 0.186 | -0.17 (-1.52, 1.21) | 0.812 |
| Tmean | 0.24 (-1.27, 1.78) | 0.756 | 0.32 (-0.43, 1.07) | 0.403 | 0.52 (-1.52, 2.60) | 0.621 |
| RH | -0.02 (-0.06, 0.02) | 0.398 | -0.01 (-0.03, 0.01) | 0.464 | -0.01 (-0.06, 0.05) | 0.812 |
| O_3_ | -0.04 (-0.44, 0.36) | 0.850 | -0.03 (-0.26, 0.20) | 0.796 | 0.33 (-0.24, 0.90) | 0.262 |
| Tmax | 0.19 (-1.23, 1.62) | 0.799 | 0.35 (-0.34, 1.05) | 0.320 | 0.29 (-1.63, 2.25) | 0.767 |
| RH | -0.02 (-0.05, 0.02) | 0.326 | -0.01 (-0.03, 0.01) | 0.252 | -0.01 (-0.06, 0.04) | 0.658 |
| O_3_ | -0.05 (-0.44, 0.35) | 0.808 | -0.01 (-0.24, 0.21) | 0.919 | 0.37 (-0.20, 0.94) | 0.209 |
| Tmin | 0.43 (-1.22, 2.10) | 0.613 | 0.23 (-0.59, 1.06) | 0.583 | -0.15 (-2.35, 2.10) | 0.894 |
| RH | -0.02 (-0.05, 0.02) | 0.380 | -0.01 (-0.03, 0.01) | 0.368 | -0.01 (-0.06, 0.04) | 0.720 |
| O_3_ | -0.04 (-0.44, 0.35) | 0.831 | -0.03 (-0.25, 0.20) | 0.820 | 0.34 (-0.23, 0.92) | 0.240 |
| Tmean | 0.29 (-1.29, 1.89) | 0.721 | 0.37 (-0.41, 1.16) | 0.350 | 0.12 (-2.00, 2.30) | 0.911 |
| RH | -0.02 (-0.05, 0.02) | 0.360 | -0.01 (-0.03, 1.01) | 0.423 | -0.02 (-0.07, 0.03) | 0.433 |
| NO_2_ | 0.25 (-0.61, 1.12) | 0.569 | 0.31 (-0.15, 0.78) | 0.185 | 0.57 (-0.69, 1.84) | 0.380 |
| Tmax | 0.18 (-1.18, 1.55) | 0.800 | 0.36 (-0.30, 1.03) | 0.291 | 0.72 (-1.13, 2.60) | 0.449 |
| RH | -0.02 (-0.05, 0.01) | 0.267 | -0.01 (-0.03, 0.00) | 0.146 | -0.03 (-0.07, 0.01) | 0.169 |
| NO_2_ | 0.28 (-0.59, 1.15) | 0.534 | 0.32 (-0.15, 0.79) | 0.181 | 0.58 (-0.70, 1.88) | 0.377 |
| Tmin | 0.45 (-1.16, 2.08) | 0.587 | 0.29 (-0.59, 1.06) | 0.583 | 0.49 (-1.67, 2.69) | 0.661 |
| RH | -0.02 (-0.05, 0.02) | 0.337 | -0.01 (-0.03, 0.01) | 0.308 | -0.02 (-0.07, 0.02) | 0.307 |
| NO_2_ | 0.26 (-0.61, 1.13) | 0.558 | 0.32 (-0.14, 0.78) | 0.178 | 0.59 (-0.68, 1.87) | 0.368 |
| Tmean | 0.29 (-1.23, 1.84) | 0.712 | 0.40 (-0.35, 1.15) | 0.300 | 0.68 (-1.37, 2.78) | 0.520 |
| ^1^ p-value <0.050 in bold  ^2^ per 5% increase in RH at Lag01  ^*^Adjusted for study location, season, chronic respiratory disease | | | | | | |

**Table S3.**  A table showing the main effects of relative humidity (RH), air pollution, and temperature on FeNO in German adolescents when stratified by SES.

|  | Low | | Medium | | High | |
| --- | --- | --- | --- | --- | --- | --- |
|  | Percentage change (95% CI)^2*^ | p-value^1*^ | Percentage change (95% CI)^2*^ | p-value^1*^ | Percentage change (95% CI)^2*^ | p-value^1*^ |
| RH | 0.03 (-0.04, 0.10) | 0.387 | 0.00 (-0.04, 0.03) | 0.760 | -0.01 (-0.03, 0.01) | 0.163 |
| PM_2.5_ | 0.45 (-1.32, 2.25) | 0.625 | 0.58 (-0.23, 1.41) | 0.162 | 0.01 (-0.52, 0.54) | 0.975 |
| Tmax | 1.64 (-0.91, 4.27) | 0.212 | 0.69 (-0.47, 1.86) | 0.247 | 0.11 (-0.57, 0.78) | 0.759 |
| RH | 0.01 (-0.04, 0.07) | 0.677 | -0.01 (-0.04, 0.01) | 0.341 | -0.01 (-0.03, 0.00) | 0.080 |
| PM_2.5_ | 0.51 (-1.25, 2.31) | 0.573 | 0.60 (-0.22, 1.43) | 0.150 | 0.02 (-0.52, 0.55) | 0.955 |
| Tmin | 1.54 (-1.32, 4.48) | 0.296 | 0.44 (-0.94, 1.84) | 0.537 | 0.14 (-0.66, 0.95) | 0.728 |
| RH | 0.02 (-0.04, 0.09) | 0.478 | -0.01 (-0.04, 0.02) | 0.575 | -0.01 (-0.03, 0.00) | 0.134 |
| PM_2.5_ | 0.47 (-1.30, 2.27) | 0.605 | 0.60 (-0.22, 1.42) | 0.154 | 0.01 (-0.52, 0.55) | 0.965 |
| Tmean | 1.70 (-1.08, 4.55) | 0.236 | 0.66 (-0.64, 1.99) | 0.320 | 0.15 (-0.61, 0.91) | 0.699 |
| RH | 0.03 (-0.06, 0.11) | 0.544 | -0.01 (-0.04, 0.02) | 0.570 | -0.01 (-0.03, 0.01) | 0.256 |
| O_3_ | -0.14 (-1.09, 0.81) | 0.770 | -0.12 (-0.50, 0.26) | 0.538 | 0.03 (-0.20, 0.25) | 0.809 |
| Tmax | 2.16 (-0.54, 4.95) | 0.121 | 0.82 (-0.41, 2.06) | 0.192 | 0.09 (-0.62, 0.80) | 0.802 |
| RH | 0.01 (-0.07, 0.09) | 0.883 | -0.02 (-0.05, 0.01) | 0.269 | -0.01 (-0.03, 0.01) | 0.189 |
| O_3_ | -0.05 (-0.97, 0.89) | 0.921 | -0.07 (-0.44, 0.30) | 0.727 | 0.03 (-0.19, 0.25) | 0.810 |
| Tmin | 1.95 (-1.03, 5.02) | 0.204 | 0.45 (-0.98, 1.90) | 0.540 | 0.12 (-0.72, 0.96) | 0.784 |
| RH | 0.02 (-0.06, 0.10) | 0.665 | -0.01 (-0.05, 0.02) | 0.416 | -0.01 (-0.03, 0.01) | 0.232 |
| O_3_ | -0.10 (-1.04, 0.84) | 0.828 | 0.10 (-0.47, 0.28) | 0.609 | 0.03 (-0.20, 0.25) | 0.825 |
| Tmean | 2.24 (-0.67, 5.23) | 0.135 | 0.75 (-0.61, 2.14) | 0.282 | 0.13 (-0.66, 0.93) | 0.746 |
| RH | 0.02 (-0.06, 0.09) | 0.646 | -0.01 (-0.04, 0.02) | 0.613 | -0.01 (-0.03, 0.01) | 0.159 |
| NO_2_ | 1.10 (-0.69, 2.93) | 0.232 | 0.74 (-0.13, 1.62) | 0.095 | 0.14 (-0.30, 0.59) | 0.531 |
| Tmax | 1.94 (-0.61, 4.55) | 0.140 | 0.82 (-0.34, 2.00) | 0.169 | 0.13 (-0.54, 0.81) | 0.701 |
| RH | -0.01 (-0.07, 0.05) | 0.849 | -0.02 (-0.05, 0.01) | 0.201 | -0.01 (-0.03, 0.00) | 0.073 |
| NO_2_ | 1.20 (-0.59, 3.03) | 0.192 | 0.71 (-0.16, 1.60) | 0.109 | 0.15 (-0.30, 0.61) | 0.505 |
| Tmin | 1.95 (-0.92, 4.89) | 0.187 | 0.56 (-0.84, 1.97) | 0.438 | 0.19 (-0.62, 1.01) | 0.644 |
| RH | 0.01 (-0.06, 0.08) | 0.800 | -0.01 (-0.04, 0.02) | 0.414 | -0.01 (-0.03, 0.00) | 0.128 |
| NO_2_ | 1.15 (-0.64, 2.97) | 0.212 | 0.74 (-0.13, 1.62) | 0.098 | 0.15 (-0.30, 0.60) | 0.517 |
| Tmean | 2.10 (-0.66, 4.95) | 0.140 | 0.80 (-0.51, 2.14) | 0.233 | 0.19 (-0.58, 0.95) | 0.634 |
| ^1^ p-value <0.050 in bold  ^2^ per 5% increase in RH at Lag01  ^*^Adjusted for study location, season, chronic respiratory disease | | | | | | |

**Table S4**. A table showing the main effects of relative humidity (RH), air pollution, and temperature on FeNO in German adolescents when stratified by CRD status.

|  | CRD: Yes | | CRD: No | |
| --- | --- | --- | --- | --- |
|  | Percentage change (95% CI)^2*^ | p-value^1*^ | Percentage change (95% CI)^2*^ | p-value^1*^ |
| RH | -0.03 (-0.06, 0.00) | 0.070 | 0.00 (-0.02, 0.02) | 0.791 |
| PM_2.5_ | 0.15 (-0.76, 1.08) | 0.742 | 0.24 (-0.23, 0.72) | 0.312 |
| Tmax | 0.55 (-0.65, 1.76) | 0.369 | 0.17 (-0.45, 0.79) | 0.601 |
| RH | -0.03 (-0.06, -0.01) | **0.016** | 0.00 (-0.02, 0.01) | 0.934 |
| PM_2.5_ | 0.19 (-0.73, 1.12) | 0.689 | 0.24 (-0.23, 0.72) | 0.312 |
| Tmin | 0.94 (-0.49, 2.39) | 0.197 | -0.04 (-0.77, 0.69) | 0.908 |
| RH | -0.03 (-0.06, -0.00) | **0.050** | 0.00 (-0.02, 0.02) | 0.920 |
| PM_2.5_ | 0.17 (-0.75, 1.09) | 0.721 | 0.25 (-0.22, 0.72) | 0.305 |
| Tmean | 0.79 (-0.55, 2.15) | 0.249 | 0.10 (-0.59, 0.80) | 0.771 |
| RH | -0.02 (-0.06, 0.01) | 0.256 | 0.00 (-0.02, 0.02) | 0.894 |
| O_3_ | 0.21 (-0.18, 0.60) | 0.286 | -0.09 (-0.29, 0.12) | 0.407 |
| Tmax | 0.38 (-0.87, 1.64) | 0.555 | 0.28 (-0.37, 0.94) | 0.399 |
| RH | -0.02 (-0.05, 0.01) | 0.169 | 0.00 (-0.02, 0.01) | 0.617 |
| O_3_ | 0.20 (-0.19, 0.58) | 0.311 | -0.06 (-0.26, 0.14) | 0.558 |
| Tmin | 0.75 (-0.71, 2.23) | 0.316 | 0.03 (-0.74, 0.80) | 0.946 |
| RH | -0.02 (-0.05, 0.01) | 0.240 | 0.00 (-0.02, 0.02) | 0.747 |
| O_3_ | 0.20 (-0.19, 0.59) | 0.310 | -0.08 (-0.28, 0.13) | 0.460 |
| Tmean | 0.61 (-0.78, 2.02) | 0.390 | 0.21 (-0.52, 0.95) | 0.570 |
| RH | -0.03 (-0.06, 0.00) | 0.091 | 0.00 (-0.02, 0.02) | 0.980 |
| NO_2_ | -0.10 (-0.91, 0.72) | 0.806 | 0.51 (0.09, 0.94) | **0.017** |
| Tmax | 0.57 (-0.63, 1.78) | 0.355 | 0.26 (-0.36, 0.89) | 0.407 |
| RH | -0.03 (-0.06, 0.00) | **0.021** | 0.00 (-0.02, 0.01) | 0.650 |
| NO_2_ | -0.06 (-0.88, 0.76) | 0.879 | 0.51 (0.08, 0.94) | **0.020** |
| Tmin | 0.92 (-0.51, 2.37) | 0.208 | 0.11 (-0.63, 0.85) | 0.777 |
| RH | -0.03 (-0.06, 0.00) | 0.064 | 0.00 (-0.02, 0.02) | 0.863 |
| NO_2_ | -0.09 (-0.90, 0.73) | 0.835 | 0.51 (0.09, 0.94) | **0.018** |
| Tmean | 0.79 (-0.55, 2.16) | 0.248 | 0.23 (-0.47, 0.94) | 0.515 |
| ^1^ p-value <0.050 in bold  ^2^ per 5% increase in RH at Lag01  ^*^Adjusted for study location, season | | | | |

**Table S5.** A table showing the main effects of relative humidity (RH), air pollution, and temperature on FeNO in German adolescents when stratified by participant location.

|  | Wesel | | Munich | |
| --- | --- | --- | --- | --- |
|  | Percentage change (95% CI)^2*^ | p-value^1*^ | Percentage change (95% CI)^2*^ | p-value^1*^ |
| RH | 0.01 (-0.02, 0.03) | 0.527 | -0.02 (-0.04, 0.00) | 0.068 |
| PM_2.5_ | 0.68 (0.01, 1.35) | **0.049** | -0.14 (-0.71, 0.43) | 0.622 |
| Tmax | 0.26 (-0.62, 1.14) | 0.569 | 0.22 (-0.53, 0.98) | 0.560 |
| RH | 0.01 (-0.02, 0.03) | 0.628 | -0.02 (-0.04, -0.01) | **0.012** |
| PM_2.5_ | 0.69 (0.01, 1.36) | **0.046** | -0.13 (-0.70, 0.44) | 0.645 |
| Tmin | 0.23 (-0.75, 1.21) | 0.647 | 0.21 (-0.73, 1.16) | 0.656 |
| RH | 0.01 (-0.02, 0.03) | 0.523 | -0.02 (-0.04, 0.00) | **0.036** |
| PM_2.5_ | 0.69 (0.02, 1.36) | **0.045** | -0.14 (-0.71, 0.43) | 0.629 |
| Tmean | 0.34 (-0.60, 1.30) | 0.478 | 0.21 (-0.66, 1.09) | 0.635 |
| RH | 0.00 (-0.02, 0.03) | 0.695 | -0.02 (-0.04, 0.00) | 0.104 |
| O_3_ | 0.04 (-0.24, 0.33) | 0.771 | -0.02 (-0.28, 0.23) | 0.859 |
| Tmax | 0.21 (-0.70, 1.13) | 0.647 | 0.28 (-0.51, 1.08) | 0.483 |
| RH | 0.00 (-0.02, 0.03) | 0.797 | -0.02 (-0.05, -0.00) | **0.043** |
| O_3_ | 0.05 (-0.23, 0.34) | 0.708 | -0.02 (-0.27, 0.24) | 0.893 |
| Tmin | 0.11 (-0.88, 1.11) | 0.826 | 0.28 (-0.70, 1.28) | 0.574 |
| RH | 0.00 (-0.02, 0.03) | 0.718 | -0.02 (-0.04, 0.00) | 0.071 |
| O_3_ | 0.04 (-0.24, 0.33) | 0.770 | -0.02 (-0.28, 0.24) | 0.881 |
| Tmean | 0.26 (-0.71, 1.24) | 0.604 | 0.28 (-0.64, 1.21) | 0.549 |
| RH | 0.00 (-0.02, 0.03) | 0.889 | -0.02 (-0.04, 0.00) | 0.067 |
| NO_2_ | 0.73 (-0.16, 1.63) | 0.111 | 0.18 (-0.25, 0.61) | 0.417 |
| Tmax | 0.39 (-0.50, 1.29) | 0.394 | 0.27 (-0.48, 1.03) | 0.484 |
| RH | 0.00 (-0.02, 0.02) | 0.848 | -0.02 (-0.04, -0.01) | **0.011** |
| NO_2_ | 0.70 (-0.19, 1.60) | 0.123 | 0.19 (-0.24, 0.63) | 0.381 |
| Tmin | 0.30 (-0.69, 1.30) | 0.555 | 0.32 (-0.63, 1.28) | 0.508 |
| RH | 0.00 (-0.02, 0.02) | 0.970 | -0.02 (-0.04, -0.00) | **0.036** |
| NO_2_ | 0.73 (-0.16, 1.64) | 0.108 | 0.18 (-0.25, 0.61) | 0.403 |
| Tmean | 0.45 (-0.52, 1.42) | 0.363 | 0.29 (-0.59, 1.17) | 0.524 |
| ^1^ p-value <0.050 in bold  ^2^ per 5% increase in RH at Lag01  ^*^Adjusted for study season, chronic respiratory disease | | | | |
